# (An) Insight into didactics in the health professions

**DOI:** 10.3205/zma001655

**Published:** 2023-11-15

**Authors:** Ursula Walkenhorst

**Affiliations:** 1 Universtität Osnabrück, FB 8 – Humanwissenschaften, Institut für Gesundheitsforschung und Bildung (IGB), Abteilung Didaktik der Humandienstleitungsberufe, Osnabrück, Germany

## Editorial

The DACH Association for Medical Education (GMA) aims to support the interprofessional dialogue between the health professions. In doing so, it pursues the goal that the professions learn more about each other in order to learn from each other. This also includes the exchange about the different didactic approaches in the respective training and study programs. The occupational field of health and the development of one or more related didactics represent a broad field of action and research from an educational science perspective. In the discussion about didactics, different perspectives influence the interdisciplinary exchange. First of all, there is the question of the target group of didactics. In the occupational field of health, educational discourses take place on

1. the vocational school level,
2. the level of didactics in higher education,
3. the level of specific occupational groups as well as
4. the level of the general school system, a level that has been less discussed so far, as a potential school subject “health”.

On each level there are different terminologies for the respective didactics, which have to be taken into account. For example, the terms vocational didactics, didactics of vocational specialization, or vocational field didactics exist for the vocational context [1]. In the context of university didactics, the terms subject-related university didactics of health or university didactics of applied health sciences are used ([2], [3] among others). This is used, among other things, in primary qualifying courses of study in the context of the academization of the health professions. Medical didactics can also be located here, since it addresses students and is thus oriented toward higher education (e.g., [4]). Approaches specific to professions/occupational groups can be found in the sense of “subject didactics” in nursing (nursing didactics), among others [5].

Common is that didactics have taken a recognizable development through science and research and are applied on the different levels. In addition to research, the development of the different didactics is promoted by the work in various (professional) associations and working groups. In addition, efforts to think about a common didactics of health have concretized in recent years [6]. 

Looking at the multiple strands that accompany the discussion on the development of a possible common didactics of health, questions and issues arise that need to be discussed. Among other things, what is the relevance of the existence of a scientific discipline? The health professions have developed only rudimentarily in disciplinary terms alongside medicine with its long tradition in science. Only the academization of some professions in health care have made the questions about one or more didactics and thus about an independent disciplinary development virulent. A common didactics of health would have to have the claim to understand the common object, namely health, in its different characteristics and to shape the professional field of health from an interdisciplinary perspective. 

In order to discuss these questions, it is necessary, within the framework of a scientific development, to collect, sift and compare the different didactic approaches in the professional field of health. Furthermore, it is important to analyze the curricula, competence catalogs and professional qualification frameworks of health-related study programs (including medicine) with regard to their specifics and commonalities. Based on existing research results, the development of research approaches and instruments on the way to the establishment of an own research methodology in the field of didactics of health would have to be examined prospectively. This also presupposes a structural development, i.e. the promotion of interdisciplinary exchange between the various actors at conferences and congresses as well as the expansion of interprofessional working groups in the field of health didactics teaching and research.

This volume exemplifies the variety of didactic and didactic-related topics and highlights results from the field of blended learning [7], young scientists [8], NKLM 2.0 [9], of digital problem-based learning [10], gene-based discrimination [11], resilience status of dental students [12], and students’ expectations about their professional career [13]. 

## Competing interests

The author declares that she has no competing interests.
